# Clinical Characteristics and Outcomes of Primary Breast Lymphoma: The Cleveland Clinic Experience

**DOI:** 10.7759/cureus.8611

**Published:** 2020-06-14

**Authors:** Tariq Kewan, Fahrettin Covut, Ramsha Ahmed, Abdo Haddad, Hamed Daw

**Affiliations:** 1 Internal Medicine, Cleveland Clinic - Fairview Hospital, Cleveland, USA; 2 Hematology and Oncology, Cleveland Clinic - Fairview Hospital, Cleveland, USA

**Keywords:** primary breast lymphoma, incidence and prognosis, non-hodgkin's lymphoma

## Abstract

Introduction

Primary breast lymphoma (PBL) is a rare malignancy that accounts for less than 0.5% of all breast malignancies.

Materials and Methods

We retrospectively analyzed 36 PBL patients to report the clinical characteristics and outcomes of patients with indolent and aggressive histologic subtypes.

Results

Thirteen (36%) patients had aggressive and 23 (64%) had indolent PBL. Marginal zone lymphoma was the most common histologic subtype (33%). Stage IE, IIE, and IV disease were seen in 27 (75%), six (17%), and three (8%) patients, respectively. Patients with aggressive PBL more often presented with a breast lump and/or B symptoms (unexplained weight loss, fever, night sweats) (78% vs. 31%, p = 0.005). Commonly used treatment modalities for aggressive vs. indolent PBL were chemotherapy alone (23% vs. 26%, p = 0.8), chemoradiotherapy (46% vs. 9%, p = 0.009), radiotherapy alone (15% vs. 22%, p = 0.6), and observation (0% vs. 26%, p = 0.07), respectively. The five-year overall survival (OS) and progression-free survival (PFS) of PBL patients were 82% (95% CI: 67 - 100) and 63% (95% CI: 45 - 89), respectively. The five-year OS of patients with aggressive vs. indolent PBL were 92% (95% CI: 77 - 100) vs. 80% (95% CI: 63 - 100), respectively (p = 0.6). The five-year OS of patients who received > 1, 1, and 0 treatment modalities were 92% (95% CI: 77 - 100), 86% (95% CI: 63 - 100), and 53% (95% CI: 21 - 100), respectively.

Conclusion

In our cohort, the higher utilization of chemoradiotherapy in aggressive PBL was able to overcome the worse prognosis of these patients. At least one treatment modality should be considered in patients with indolent PBL, given that observation alone was associated with a poor prognosis.

## Introduction

Primary breast lymphoma (PBL) is a rare malignancy that accounts for less than 3% of extranodal lymphoma, 1% of non-Hodgkin's lymphoma (NHL), and 0.5% of all breast malignancies [1-3]. PBL was first described in 1972 by Wiseman and Liao as breast tissue infiltration by lymphoma with or without regional lymph node involvement in patients without prior nodal or extranodal lymphoma at the time of diagnosis [4]. This definition was reviewed in 1990 by Hugh et al. [5]. Patients presenting with lymphoma of regional lymph nodes and bilateral breast involvement were also included in the definition of PBL [6]. PBL affects female patients in > 95% of the cases and involves the right breast more than the left. Diffuse large B-cell lymphoma (DLBCL) is the most commonly reported histological subtype [1-2, 7-9]. Marginal zone lymphoma, follicular lymphoma, and mucosa-associated lymphoma are not as common as DLBCL [1-2, 10-11]. In addition, anaplastic large T-cell lymphoma (ALCL) and Hodgkin’s lymphoma represent a very small proportion of PBL. ALCL is usually localized and presents as a mass or an effusion. Recently, other PBL subtypes have been reported after plastic surgery procedures [2, 12].

Clinically, PBL can be diagnosed as an incidental finding on mammogram or patients can present with breast swelling and B symptoms (unexplained weight loss, fever, night sweats). Mammographic features of PBL are distinct from invasive breast cancer. The role of breast ultrasound and magnetic resonance imaging in PBL diagnosis is not well established yet [7-8, 13]. Retrospective studies reported no benefit of radical mastectomy compared to partial mastectomy or biopsy in terms of overall survival (OS) and progression-free survival (PFS) rates. A combination of chemoradiotherapy has been shown to increase complete remission rates compared to chemotherapy or radiotherapy alone [1, 7, 13-19]. The International Extranodal Lymphoma Study Group reported that a combination of surgery, chemotherapy, and radiation to involved-field yielded the best prognosis [9, 15, 17, 19].

In view of the rarity of PBL, we hereby report the clinical characteristics and outcomes of PBL patients after different treatment modalities to contribute to defining optimal treatment strategy for this rare lymphoma subtype.

## Materials and methods

We retrospectively identified patients who were diagnosed with PBL between January 2014 and January 2019 at Cleveland Clinic Foundation. This study was approved by the Institutional Review Board of the Cleveland Clinic (#18-1303). PBL was defined as breast tissue infiltration by lymphoma with or without regional lymph node involvement in patients without prior nodal or extranodal lymphoma at the time of diagnosis [4]. We included patients with Stage IE, IIE, and IV disease and considered patients with bilateral breast involvement to have Stage IIE PBL [1-2, 5, 9]. The World Health Organization histologic classification was used for histologic classification of PBL [20]. Clinical variables, including age at diagnosis, presenting symptoms, performance status at diagnosis, history of breast implant, affected breast, lymphoma histologic type, stage at diagnosis, treatment modalities (surgery, radiotherapy, chemotherapy, central nervous system chemoprophylaxis), and relapse data, were collected. Aggressive PBL was defined as DLBCL, breast implant-associated ALCL, and unclassifiable B-cell lymphoma with features intermediate between DLBCL and Burkitt's lymphoma, while other histological subtypes were defined as indolent PBL.

Baseline characteristics were compared between the indolent and aggressive PBL groups using a two-sample T-test for continuous variables, Chi-square test, and Fisher’s exact test for categorical variables. OS and PFS were estimated by the Kaplan-Meier method and compared by the log-rank test. PFS was calculated from the date of diagnosis to the date of first recurrence, progressions, or death. A p-value of less than 0.05 was considered statistically significant. All statistical calculations were made using R statistical software version 3.4.0 (R Foundation for Statistical Computing, Vienna, Austria).

## Results

A total of 36 patients with PBL were identified. Median age at diagnosis was 66 years (range: 34 - 95) and 97% of the patients were female. Of all patients, 23 (64%) had indolent lymphoma and 13 (36%) had aggressive lymphoma. The most common histologic subtypes were marginal zone lymphoma (33%), DLBCL (25%), and follicular lymphoma (19%). Only two (6%) patients had implant-associated ALCL and one (3%) patient had classic nodular sclerosing Hodgkin lymphoma. Five (14%) patients had bilateral breast involvement at the time of diagnosis. Among all patients, 27 (75%) had Stage IE, six (17%) had Stage IIE, and three (8%) had Stage IV disease at the time of diagnosis. Patients with indolent PBL were more often asymptomatic and diagnosed via screening mammograms compared to those with aggressive PBL (78% vs. 31%, p = 0.005, respectively). On the contrary, patients with aggressive PBL more often presented with a breast lump and/or B-symptoms compared to those with indolent PBL (69% vs. 22%, p = 0.005, respectively). Only two (6%) patients in the entire cohort presented with B symptoms, along with a breast lump, at the time of diagnosis. Table 1 summarizes the baseline and treatment characteristics of the patients. 

**Table 1:** Patient and Treatment Characteristics BCL-U: B-cell lymphoma - unclassifiable; BIA-ALCL: breast implant-associated anaplastic large T-cell lymphoma; CLL/SLL: chronic lymphocytic leukemia/small lymphocytic leukemia; DLBCL: diffuse large B-cell lymphoma; ECOG: Eastern Cooperative Oncology Group

Characteristics	All Patients n (%)	Indolent n (%)	Aggressive n (%)	P-value
Age (year), median (range)	66 (34 – 95)	66 (34 – 83)	67 (49 – 95)	0.46
Age > 65	21 (58.3)	12 (52.2)	9 (69.2)	0.48
Male	1 (2.8)	0 (0)	1 (7.7)	0.36
Female	35 (97.2)	23 (100)	12 (92.3)	0.47
White ethnicity	35 (97.2)	22 (95.7)	13 (100)	1
African American	1 (2.8)	1 (4.3)	0 (0)	0.65
Clinical presentation at diagnosis				
Abnormal screening mammogram	22 (61.1)	18 (78.3)	4 (30.8)	0.005
Breast lump/swelling	14 (38.9)	5 (21.7)	9 (69.2)	0.005
B-symptoms	2 (5.6)	1 (4.3)	1 (7.7)	0.67
ECOG performance score at diagnosis				0.31
0 or 1	34 (94.4)	22 (95.7)	12 (92.3)	
2	1 (2.8)	1 (4.3)	0 (0)	
3	1 (2.8)	0 (0)	1 (7.7)	
Laterality of tumor				0.69
Right	14 (38.9)	9 (39.1)	5 (38.5)	
Left	17 (47.2)	10 (43.5)	7 (53.8)	
Bilateral	5 (13.9)	4 (17.4)	1 (7.7)	
Stage at diagnosis				0.74
IE	27 (75.0)	18 (78.3)	9 (69.2)	
IIE	6 (16.7)	3 (13.0)	3 (23.1)	
IV	3 (8.3)	2 (8.7)	1 (7.7)	
Histologic type				–
Marginal zone	12 (33.3)	12 (52.2)	0 (0)	
Diffuse large B cell lymphoma	9 (25.0)	0 (0)	9 (69.2)	
Follicular lymphoma	7 (19.4)	7 (30.4)	0 (0)	
CLL/SLL	3 (8.3)	3 (13.0)	0 (0)	
BIA-ALCL	2 (5.6)	0 (0)	2 (15.4)	
BCL-U (DLBCL or Burkitt lymphoma)	2 (5.6)	0 (0)	2 (15.4)	
Nodular sclerosing Hodgkin lymphoma	1 (2.8)	1 (4.3)	0 (0)	
Treatment types				
Observation	6 (16.7)	6 (26.1)	0 (0)	0.07
Chemotherapy only	9 (25.0)	6 (26.1)	3 (23.1)	0.84
Radiotherapy only	7 (19.4)	5 (21.7)	2 (15.4)	0.64
Surgery only	2 (5.6)	1 (4.3)	1 (7.7)	1
Surgery/chemotherapy	2 (5.6)	1 (4.3)	1 (7.7)	1
Chemotherapy/radiotherapy	8 (22.2)	2 (8.7)	6 (46.2)	0.009
Surgery/radiotherapy	1 (2.8)	1 (4.3)	0 (0)	1
Surgery/chemotherapy/radiotherapy	1 (2.8)	1 (4.3)	0 (0)	1

Commonly used treatment modalities for aggressive vs. indolent PBL were chemotherapy alone (23% vs. 26%, p = 0.8), chemoradiotherapy (46% vs. 9%, p = 0.009), radiotherapy alone (15% vs. 22%, p = 0.6), and observation (0% vs. 26%, p = 0.07), respectively. Commonly used chemotherapy regimens in aggressive vs. indolent PBL were rituximab, cyclophosphamide, doxorubicin hydrochloride, vincristine (Oncovin), and prednisone (R-CHOP) (40% vs. 40%, p = 1), rituximab alone (10% vs. 30%, p = 0.58), rituximab, etoposide phosphate, prednisone, vincristine sulfate (Oncovin), cyclophosphamide, and doxorubicin hydrochloride (hydroxydaunorubicin) (R-EPOCH) (30% vs. 0%, p = 0.21), and rituximab/bendamustine (0% vs. 20%, p = 0.47), respectively (Table 2). Surgery was performed for six (17%) patients; three underwent a lumpectomy, two underwent implant removal with complete capsulectomy for implant-associated ALCL, and one underwent a partial mastectomy. Single and a combination of more than one treatment modality were used for six (46%) aggressive vs. 12 (52%) indolent (p = 0.7) and seven (54%) aggressive vs. five (22%) indolent (p = 0.05) PBL cases, respectively. Prophylactic intrathecal chemotherapy was used in one patient with Stage IIE bilateral PBL and this patient didn’t have any central nervous system (CNS) relapse.

**Table 2:** First-Line Chemotherapy Regimens ABVD: Adriamycin (doxorubicin), bleomycin, vinblastine, dacarbazine; CHOEP: cyclophosphamide, doxorubicin, etoposide, vincristine, prednisone; CODOX: cyclophosphamide, vincristine, doxorubicin; R-CHOP: rituximab, cyclophosphamide, doxorubicin, vincristine, prednisone; R-EPOCH: rituximab, etoposide phosphate, prednisone, vincristine sulfate (Oncovin), cyclophosphamide, and doxorubicin hydrochloride (hydroxydaunorubicin)

Chemotherapy regimens	All Patients n (%)	Indolent n (%)	Aggressive n (%)	P-value
R-CHOP	8 (40)	4 (40)	4 (40)	1
Rituximab	4 (20)	3 (30)	1 (10)	0.58
R-EPOCH	3 (15)	0 (0)	3 (30)	0.21
Rituximab/bendamustine	2 (10)	2 (20)	0 (0)	0.47
ABVD	1 (5)	1 (10)	0 (0)	1
CHOEP	1 (5)	0 (0)	1 (10)	1
CODOX	1 (5)	0 (0)	1 (10)	1

**Figure 1:**
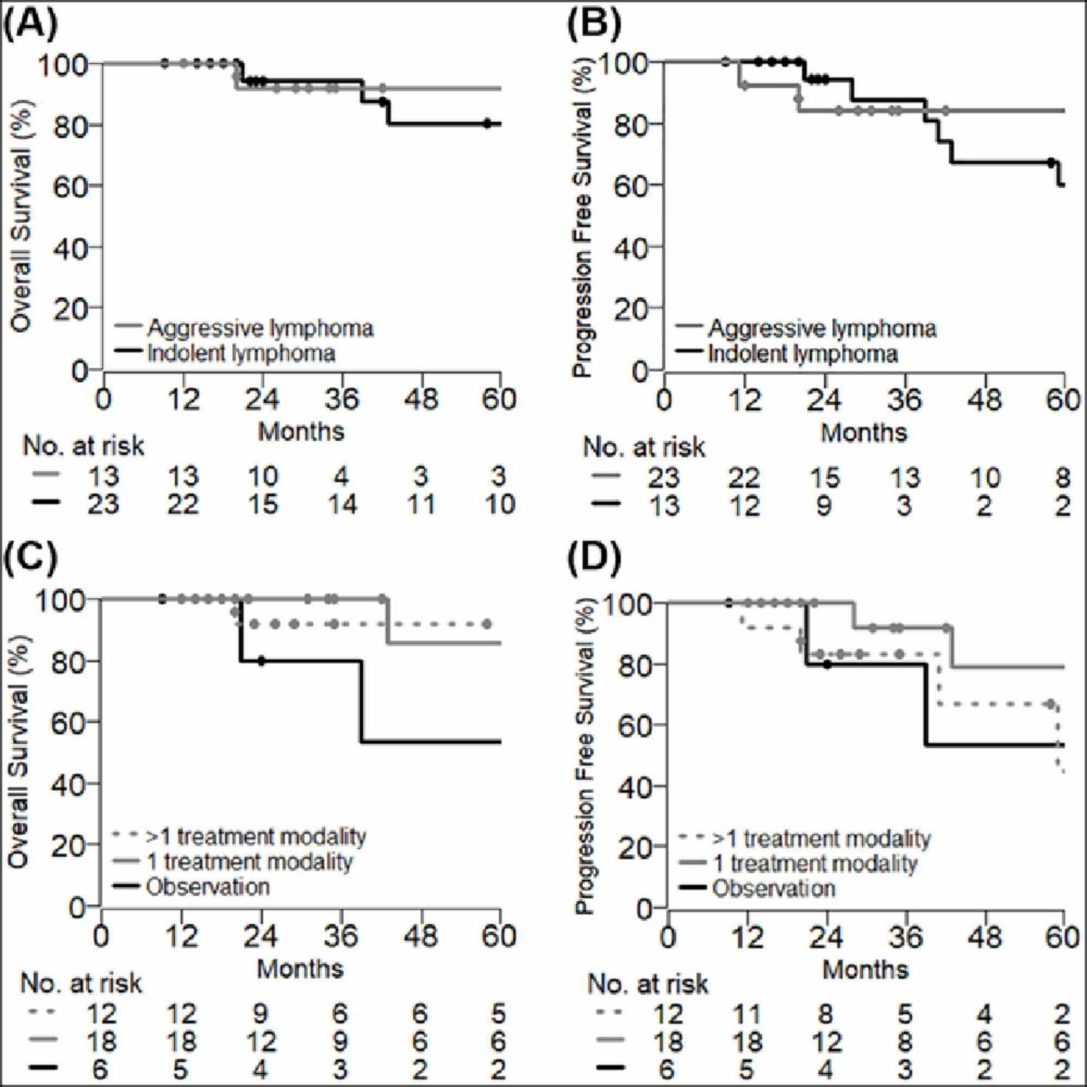
Overall survival and relapse-free survival of patients with primary breast lymphoma (A - B) Stratified based on histologic subtype; (C - D) Stratified based on number of treatment modalities

The median follow-up of these patients was 37 months. Among six (17%) patients who had disease relapse, only one patient with Stage IE primary breast DLBCL had CNS relapse, and another patient died. Six (17%) patients died, of which all were above 65 years old at the time of diagnosis; three were observed for indolent PBL, one received radiotherapy for indolent PBL, and two patients received radiotherapy alone and chemoradiotherapy for aggressive PBL. The five-year OS and PFS of PBL patients were 82% (95% CI: 67 - 100) and 63% (95% CI: 45 - 89), respectively. Five-year OS and PFS of patients with aggressive vs. indolent PBL were 92% (95% CI: 77 - 100) vs. 80% (95% CI: 63 - 100), p = 0.6, and 84% (95% CI: 66 - 100) vs. 60% (95% CI: 39 - 91), p = 0.9, respectively (Figure 1A-B). The five-year OS of patients who received a combination of more than one treatment modality for PBL was 92% (95% CI: 77 - 100) compared to 86% (95% CI: 63 - 100), p = 0.9 and 53% (95% CI: 21 - 100), p = 0.07 for patients who were treated with a single treatment modality and observed, respectively (Figure 1C).

## Discussion

The typical clinical presentation of PBL is with a solitary breast lump in female patients between 50 and 70 years of age [1, 8]. Similarly, 39% of the patients presented with a breast lump, and the median age of the patients was 66 years in our study. Although DLBCL was the most common histological subtype in previous studies, marginal zone lymphoma was the most common histological subtype in our cohort and only 25% of our patients had DLBCL [1, 3, 5-6]. The five-year OS of PBL patients in the present study was similar to what has been previously reported [1, 9, 19, 21]. Tumor size, laterality, and histological lymphoma subtype were not prognostic indicators in our study and the majority of the prior studies [1, 4, 6-9, 21]. Although Zu et al. reported Ann Arbor's clinical stage and B symptoms as independent prognostic factors for OS and PFS, they were not predictors of OS or PFS in our cohort [9]. 

In a retrospective study by Ryan et al., treatment with anthracycline-containing chemotherapy and radiotherapy prolonged the OS [19]. Prior studies showed that radiotherapy for Stage I and chemotherapy for Stage II PBL patients prolonged both OS and PFS [22-23]. To date, only one randomized clinical trial was performed for PBL patients in which chemoradiotherapy prolonged OS and event-free survival more than chemotherapy or radiotherapy alone [24]. In our cohort, aggressive PBL cases were more often treated with chemoradiotherapy compared to indolent PBL cases. Importantly, three of six (50%) patients with indolent PBL who were observed without any treatment died within four years of diagnosis. This finding suggests that the use of at least a single treatment modality should be considered, even for patients with indolent PBL. However, there was no statistically significant difference between the different treatment modalities, maybe in part due to the important limitations of our study, such as the single-center retrospective study design with a small number of patients.

Rituximab is routinely implemented in chemotherapy for primary breast DLBCL patients [1, 15-16, 25]. The use of rituximab and radiotherapy in primary breast DLBCL has been found to improve OS and reduce relapse rates [3, 16, 18]. On the other hand, Hosein et al. noted in a retrospective study that rituximab use was not associated with any survival benefit [21]. To date, no randomized clinical trials were performed to assess the efficacy of rituximab for the treatment of PBL. In the present study, 85% of the patients treated with chemotherapy and 47% of the whole cohort received rituximab.

The overall rate of CNS relapse in patients with PBL has been reported to be around 8% [24]. Prophylactic intrathecal chemotherapy is controversial and should only be considered in high-risk patients, such as patients with Stage IIE disease and aggressive lymphoma histology [1, 13, 22]. CNS relapse was less common in our cohort with a rate of 3%.

## Conclusions

In this cohort of patients with PBL, marginal zone lymphoma was the most common histological subtype and mortality was seen only in elderly patients regardless of the histologic subtype. Higher utilization of chemoradiotherapy in aggressive PBL was able to overcome the worse prognosis of these patients. At least one treatment modality should be considered in patients with indolent PBL given that observation alone was associated with a poor prognosis.
